# Modulation of activation and inactivation by Ca^2+^ and 2-APB in the pore of an archetypal TRPM channel from *Nematostella vectensis*

**DOI:** 10.1038/s41598-017-07652-4

**Published:** 2017-08-03

**Authors:** Frank J. P. Kühn, Winking Mathis, Kühn Cornelia, Daniel C. Hoffmann, Andreas Lückhoff

**Affiliations:** Institute of Physiology, Medical Faculty, RWTH Aachen, D52057 Aachen, Germany

## Abstract

The archetypal TRPM2-like channel of the sea anemone *Nematostella vectensis* is gated by ADPR like its human orthologue but additionally exhibits properties of other vertebrate TRPM channels. Thus it can help towards an understanding of gating and regulation of the whole subfamily. To elucidate further the role of Ca^2+^ as a co-factor of ADPR, we exploited 2-aminoethyl diphenylborinate (2-APB), previously shown to exert either inhibitory or stimulatory effects on diverse TRPM channels, or both in a concentration-dependent manner. 2-APB in high concentrations (1 mM) induced large, non-inactivating currents through *nv*TRPM2. In lower concentrations (≤0.5 mM), it prevented the fast current inactivation typical for *nv*TRPM2 stimulated with ADPR. Both these effects were rapidly reversed after wash-out of 2-APB, in contrast to a considerable lag time of their onset. A detailed analysis of *nv*TRPM2 mutants with modified selectivity filter or reduced ADP-ribose sensitivity revealed that the actions of 2-APB depend on its access to the pore which is enhanced by channel opening. Moreover, access of Ca^2+^ to the pore is decisive which again depends on the open state of the channel. We conclude that separate regulatory processes by Ca^2+^ on the pore can be discriminated with the aid of 2-APB.

## Introduction

The sea anemone *Nematostella vectensis* is frequently used as a model organism for studies on the evolution of the nervous system^1^ and the immune system^2^. In its genome, only one type of TRPM channel is present. This has been classified as TRPM2 orthologue (*nv*TRPM2) because it contains the characteristic NUDT9 homology domain (NUDT9H) within its C-terminus^3^. However, *nv*TRPM2 shows a long extracellular loop between the transmembrane segments S1 and S2 which is absent in *h*TRPM2 but well conserved e.g. in *h*TRPM3^4^. This observation is in line with the hypothesis that the TRPM2-like channel of protists and basal metazoans represents an archetypal TRPM channel^3^. Thus, *nv*TRPM2 possibly has model character for the whole TRPM subfamily rather than a single member; in a general sense, studies on *nv*TRPM2 may reveal insights into principal as well as specific properties of human TRPM channels.

During evolution, the progenitors of cnidarians and of vertebrates have separated an estimated time of 800 million years ago^5^. In spite of this long time for a divergent development, *nv*TRPM2 and *h*TRPM2 are both activated by ADPR, which is not known as a stimulus for any other ion channel^4^. Even more surprising was our recent finding that the mechanisms by which this unique stimulus leads to channel activation are strikingly different^6^. In *h*TRPM2, the enzymatically inactive NUDT9H domain specifically binds ADPR and thereby initiates channel gating^7–10^. In *nv*TRPM2, the removal of the NUDT9H domain leaves the ADPR sensitivity of the channel intact^6^. We have provided evidence that in *nv*TRPM2, the NUDT9H domain regulates the intracellular availability of ADPR by its enzymatic activity, whereas a further, yet un-identified interaction site for ADPR is present elsewhere in the channel protein^6^.

Gating of TRPM channels and, especially, of TRPM2 is quite complex and is not easily explained by simple models. In particular, the role of Ca^2+^ as a co-agonist of *h*TRPM2 and *nv*TRPM2 is poorly understood. In both cases, Ca^2+^ enters and permeates the pore and must be present at least on one side of the cell membrane to allow channel activation^4, 11–13^. Additionally, Ca^2+^ is involved in the delayed inactivation process of *h*TRPM2^12^, which can be modified by certain point mutations within the channel pore^14, 15^. Thus, several regulatory processes take place simultaneously, modulated by Ca^2+^ at several points, and these processes can hardly be separated and discriminated because of the lack of suitable experimental tools.

To get further insights into the gating mechanism of the archetypal TRPM channel from *Nematostella vectensis*, we tested the effect of 2-aminoethyl diphenylborinate (2-APB), a non-specific TRP channel modulator, which has also widely been used as a pharmacological tool to explore intracellular Ca^2+^ signaling^16–18^. 2-APB may exert either activating or inhibitory functions on closely related TRPM channels (e.g. TRPM6 vs. TRPM7^19^, or human TRPM7 vs. zebrafish TRPM7^20^). Moreover, it may have both of these functions on the same ion channel, dependent on the applied concentration (e.g. *h*TRPM7^19^). On the human TRPM2 orthologue as well as on *h*TRPM8, the closest relatives within the TRPM family, 2-APB acts exclusively as a reversible inhibitor^21–23^. The molecular basis that underlies the discrepant effects of 2-APB on TRPM channels has not been investigated. We hypothesized that these are related to a specific triplet of amino acid residues within the proximal pore loop that exists in two principal versions within the TRPM family^3^. The triplet glutamine-isoleucine-proline (QIP) in *h*TRPM2 or glutamine-valine-proline (QVP) in *h*TRPM8 results in a low selectivity for Ca^2+^, whereas the motif glutamate-valine-tyrosine (EVY) present in *h*TRPM6 and *h*TRPM7, or glutamate-valine-phenylalanine (EVF) in *h*TRPM3, creates a high permeability for Ca^2+^. Consequently, the pore signatures have been associated with the selectivity filter of the channel pore^3^. The corresponding motif ELF assigns *nv*TRPM2 to the more Ca^2+^ selective TRPM channels^3^. It is striking that 2-APB at high concentrations stimulates vertebrate TRPM channels with high permeability for Ca^2+^, (e.g. TRPM6, TRPM7)^19, 20^ whereas it uniformly inhibits those which poorly discriminate between monovalent and divalent cations (e.g. TRPM2, TRPM8)^21–23^. An exemption may be the fairly Ca^2+^-selective TRPM3, which was inhibited by 2-APB, but higher drug concentrations were not studied^24^.

Here we demonstrate that indeed *nv*TRPM2 is activated by high concentrations of 2-APB and that the activation is lost after mutating the ELF motif to QLP. But rather than simply constituting an activator or inhibitor dependent on the pore signature, 2-APB moreover proved to abrogate the rapid current inactivation that is characteristic for *nv*TRPM2 under stimulation with ADPR. Since activation as well as inactivation of *nv*TRPM2 is Ca^2+^-dependent, the present study establishes 2-APB as a valuable tool by which diverse modulatory processes by Ca^2+^ within the channel pore can be disentangled. It is hoped that these and further ongoing studies will help towards an understanding of some aspects of TRPM channel regulation in an integrative sense which applies to the whole family, under consideration of specific common and divergent structural and functional properties of individual members.

## Results

### Specific effects of 2-APB on activation and inactivation of wild-type *nv*TRPM2

**Figure 1:**
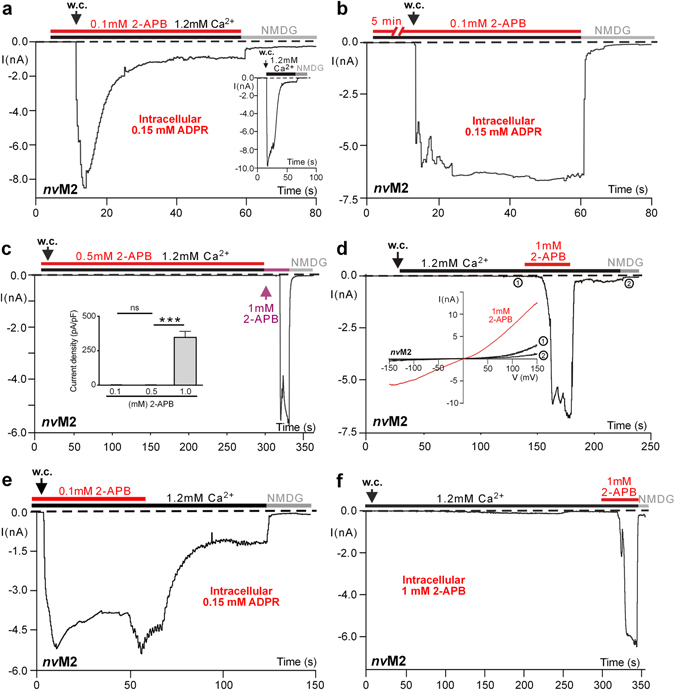
Effects on activation and inactivation of 2-APB on wild-type *nv*TRPM2 in patch-clamp experiments. (**a**) 2-APB does not inhibit ADPR-induced currents. 2-APB (0.1 mM) was added to the standard bath containing 1.2 mM Ca^2+^ just before reaching whole-cell configuration (w.c.). ADPR (0.15 mM) was infused into the cell through the patch pipette together with 1 µM Ca^2+^. As in the control experiment (inset), an immediate current activation was induced by ADPR, followed by a rapid inactivation to background current levels. Substitution of external Na^+^ with the impermeable cation NMDG blocks the remaining currents. (**b**) 2-APB inhibits current inactivation. Same as in panel a, but the compound (0.1 mM) was pre-incubated for 5 min prior to the current recordings. A plateau-like current was induced by ADPR that was later blocked by NMDG. (**c**) In the absence of ADPR, 2-APB activates *nv*TRPM2 only at a high concentration. 2-APB was first applied at a concentration of 0.5 mM and then at 1 mM. Only the highest concentration induced a non-inactivating current that was blocked by NMDG. Statistics of these experiments are shown in the inset. Asterisks indicates significant differences (***P < 0.001; Student’s t-test, *n* = 4–8). Error bars are s.e. (**d**) Current activation by 2-APB occurs in a delayed manner but is rapidly reversed after wash-out. Note the delay until the current increases with fast kinetics, resulting in a fairly constant level, whereas removal of 2-APB resulted in an almost immediate abolition of the current. The inset shows voltage ramps taken during various times of the experiment, as indicated. (**e**) ADPR-induced currents do not inactivate in the presence of 2-APB and the inhibition of current inactivation is rapidly reversed after wash-out. Note that the current decline that results after removal of 2-APB occurs almost immediately and with the kinetics characteristic for ADPR stimulation. (**f**) 2-APB acts from the outside but not from the inside. 2-APB was present in the pipette solution (1 mM) but the typical currents were induced only after additional application to the bath. All experiments were repeated at least three times confirming the results.

Since 2-APB is an established inhibitor of several Ca^2+^-permeable cation channels including *h*TRPM2, we first studied its effects as a potential inhibitor of *nv*TRPM2 (Fig. 1). After a short (30 s) pre-incubation with standard bath solution containing 2-APB (0.1 mM), a cell transfected with *nv*TRPM2 was brought into the whole-cell configuration of the patch-clamp technique (Fig. 1a). The cell was infused with a pipette solution containing 150 µM ADPR and 1 µM Ca^2+^ which allows rapid stimulation of the channels. The currents of *nv*TRPM2 induced by ADPR typically show fast activation and inactivation kinetics^4^. Large inward currents developed instantaneously in controls (Fig. 1a inset) as well as in cells pre-incubated with 2-APB with no apparent difference. When we expanded the time of pre-incubation with 2-APB (0.1 mM) to 5–10 minutes, we did not observe any indication of current inhibition either. In the contrary, 2-APB prevented the characteristic fast inactivation of ADPR-induced currents of *nv*TRPM2 and led to a sustained, plateau-like current. Such currents were readily blocked by the large cation NMDG (Fig. 1b). At a concentration of 0.5 mM, the effect of 2-APB on the inactivation required less time of pre-incubation (*n* = 5) but no effect on unstimulated cells was observed (Fig. 1c inset).

However, when 2-APB was used at higher concentrations (1 mM), the compound induced large currents by itself, without ADPR present in the intracellular (pipette) solution (Figs 1c and d). Again, these currents were sustained and could be rapidly and fully reversed by superfusion of the cells with standard bath solution without 2-APB. Importantly, the onset of 2-APB dependent currents showed a characteristic delay which was in the range of 10–30 s. Subsequently, the currents increased rapidly to reach several nanoamperes and remained constant (Fig. 1d). The I/V relation of 2-APB stimulated currents shows a reversal potential near 0 mV indicating no discrimination between monovalent and divalent cations (Fig. 1d inset). Quite in contrast to the slow onset of 2-APB effects, they were rapidly reversed in an almost complete manner by wash-out of the substance (Fig. 1d). Similarly, inactivation of ADPR-induced currents in the presence of 2-APB could be immediately restored by removal of 2-APB (Fig. 1e). No effect on current development was detected when 2-APB (1 mM) was intracellularly applied via the pipette solution (Fig. 1f) which is in line with previous findings on *h*TRPM2^22^.

### Reversible current inhibition by 2-APB in the human species variant of TRPM2

**Figure 2:**
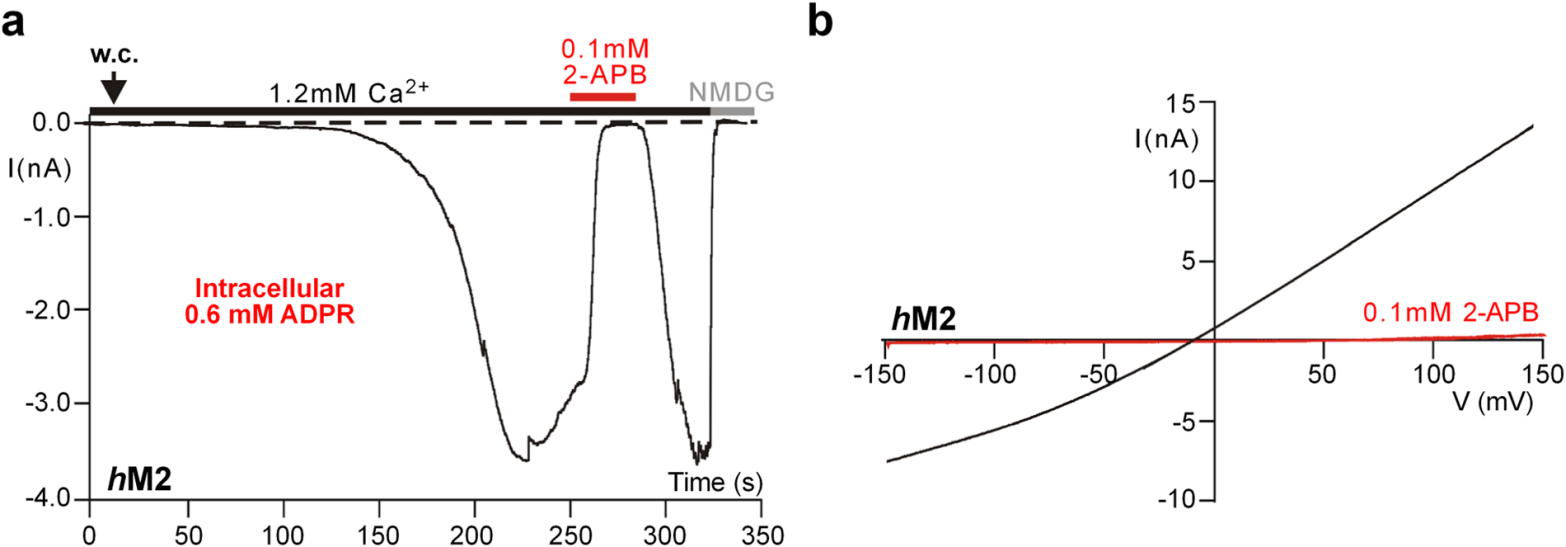
Reversible current inhibition by 2-APB in human TRPM2. (**a**) The slowly activating and hardly inactivating currents through this channel were promptly, completely, and reversibly inhibited by 2-APB (0.1 mM) applied to the standard bath. (**b**) Corresponding current-voltage relations to the experiment shown in panel a. The voltage ramps show that the inward as well as the outward current component is equally affected.

We also tested 2-APB on *h*TRPM2. In contrast to *nv*TRPM2, this channel is characterized by currents showing slow activation and inactivation kinetics after stimulation with ADPR (Fig. 2a). These currents were rapidly inhibited when the cells were superfused with a standard bath solution containing 0.1 mM 2-APB (Fig. 2a and b); the effect was completely reversible after wash-out of the inhibitor which is in good agreement with previous results^22, 23^. Thus, opposite effects of 2-APB were found on the two TRPM2 orthologues.

### Ca^2+^ represents an essential co-factor for the 2-APB-induced activation of *nv*TRPM2

**Figure 3:**
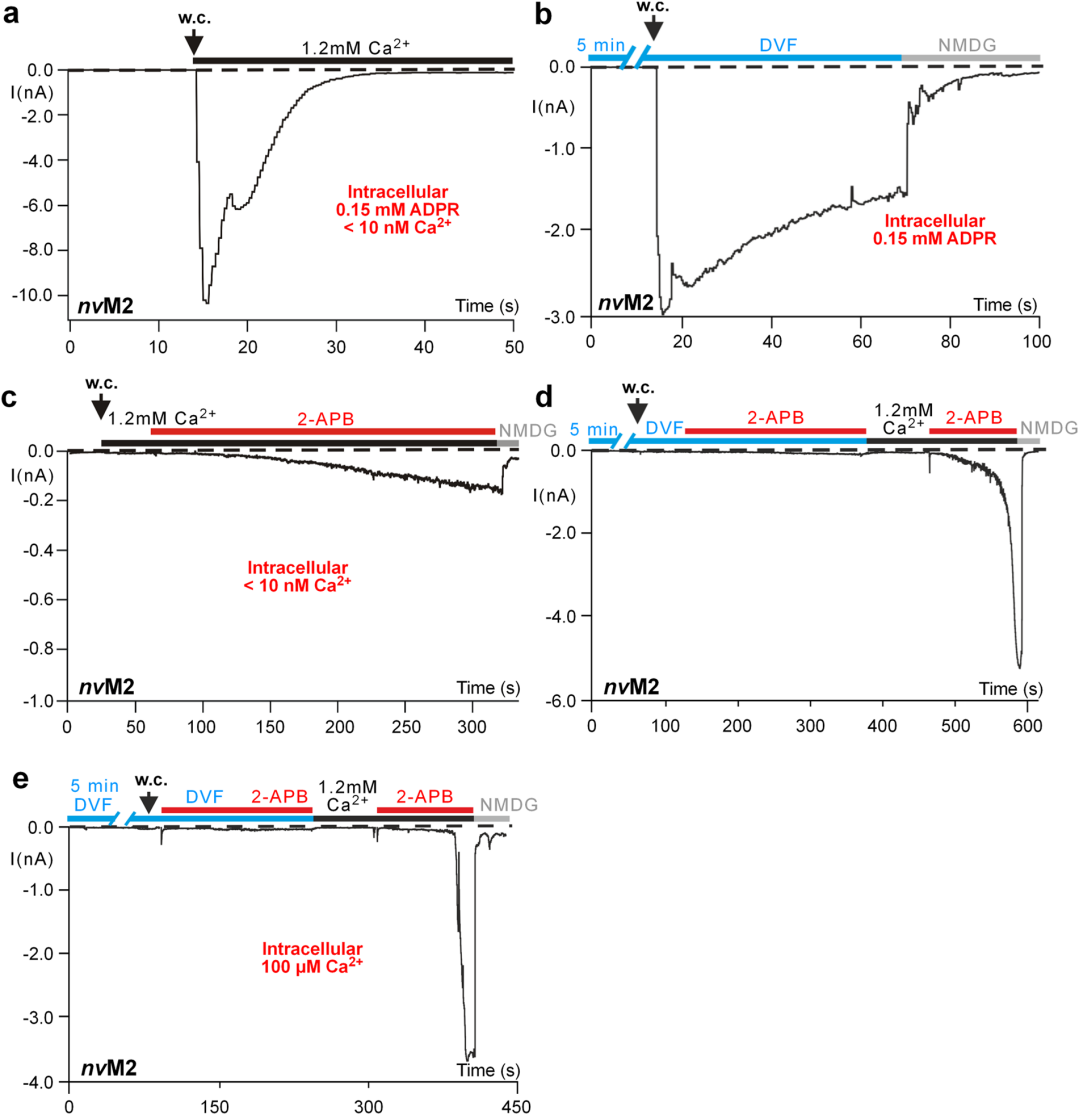
Dependence on Ca^2+^ of currents induced by ADPR or 2-APB in *nv*TRPM2. (**a**) For the stimulation with ADPR, intracellular Ca^2+^ is not required. The experiment was performed in standard bath (1.2 mM Ca^2+^) but with a pipette solution in which Ca^2+^ was buffered with EGTA to below 10 nM as indicated. (**b**) Extracellular Ca^2+^ is not required for the ADPR-dependent stimulation, but for the current inactivation. The current recording was performed with a pipette solution containing 1 µM Ca^2+^ but in the extended absence of extracellular Ca^2+^, achieved by multiple superfusion of the cells with a divalent-free solution. Note that the inactivation takes place considerably slower than under control conditions (inset Fig. 1a), reaching about 50% of the maximal current after 40 s. (**c**) Intracellular Ca^2+^ is required for the stimulation with 2-APB. Under the same Ca^2+^ conditions as in panel a (no ADPR), 2-APB (1 mM) induced only miniscule currents. (**d**) Extracellular Ca^2+^ is required for the stimulation with 2-APB. After extended exposure to DVF, 2-APB (1 mM) was without effect but did induce characteristic currents when applied a second time in the presence of a standard extracellular Ca^2+^ (1.2 mM). As in panel b, the pipette solution contains 1 µM Ca^2+^. (**e**) Strongly elevated intracellular Ca^2+^ cannot substitute extracellular Ca^2+^. Same experimental conditions as in panel d, but the intracellular solution contained 100 µM Ca^2+^. All experiments were repeated at least three times confirming the results.

Stimulation of *nv*TRPM2 as well as of *h*TRPM2 by ADPR depends on Ca^2+^ which must be present at least on one side of the cell membrane to allow channel activation^4, 12^. Removal of Ca^2+^ from one side did not largely affect ADPR-induced currents of *nv*TRPM2 (Fig. 3a). However, the inactivation of the currents was markedly slowed when a nominally divalent-free (DVF) bath solution containing 10 mM EGTA was applied (Fig. 3b). This effect was particularly pronounced when the cells were perfused with a DVF bath solution for several minutes before the recording was started. A remarkably different situation was present when 2-APB was used as activator of *nv*TRPM2. With < 10 nM Ca^2+^ in the intracellular solution, the addition of 2-APB (1 mM) to the standard bath (1.2 mM Ca^2+^) induced no more than miniscule currents that developed slowly over several minutes (Fig. 3c). In DVF bath solution (in the presence of 1 µM intracellular Ca^2+^), no significant currents were elicited by 2-APB. Only the restitution of extracellular Ca^2+^ enabled 2-APB-induced currents with the characteristic kinetics (Fig. 3d). Thus, Ca^2+^ on both sides of the pore is required for a stimulation by 2-APB. Not even a strongly elevated intracellular Ca^2+^ concentration (100 µM) was sufficient to enable 2-APB-induced currents when extracellular Ca^2+^ was absent, whereas those currents were restored when extracellular Ca^2+^ was reestablished (Fig. 3e).

### Stimulation of wild-type *nv*TRPM2 with 2-APB induces massive influx of Ca^2+^

**Figure 4:**
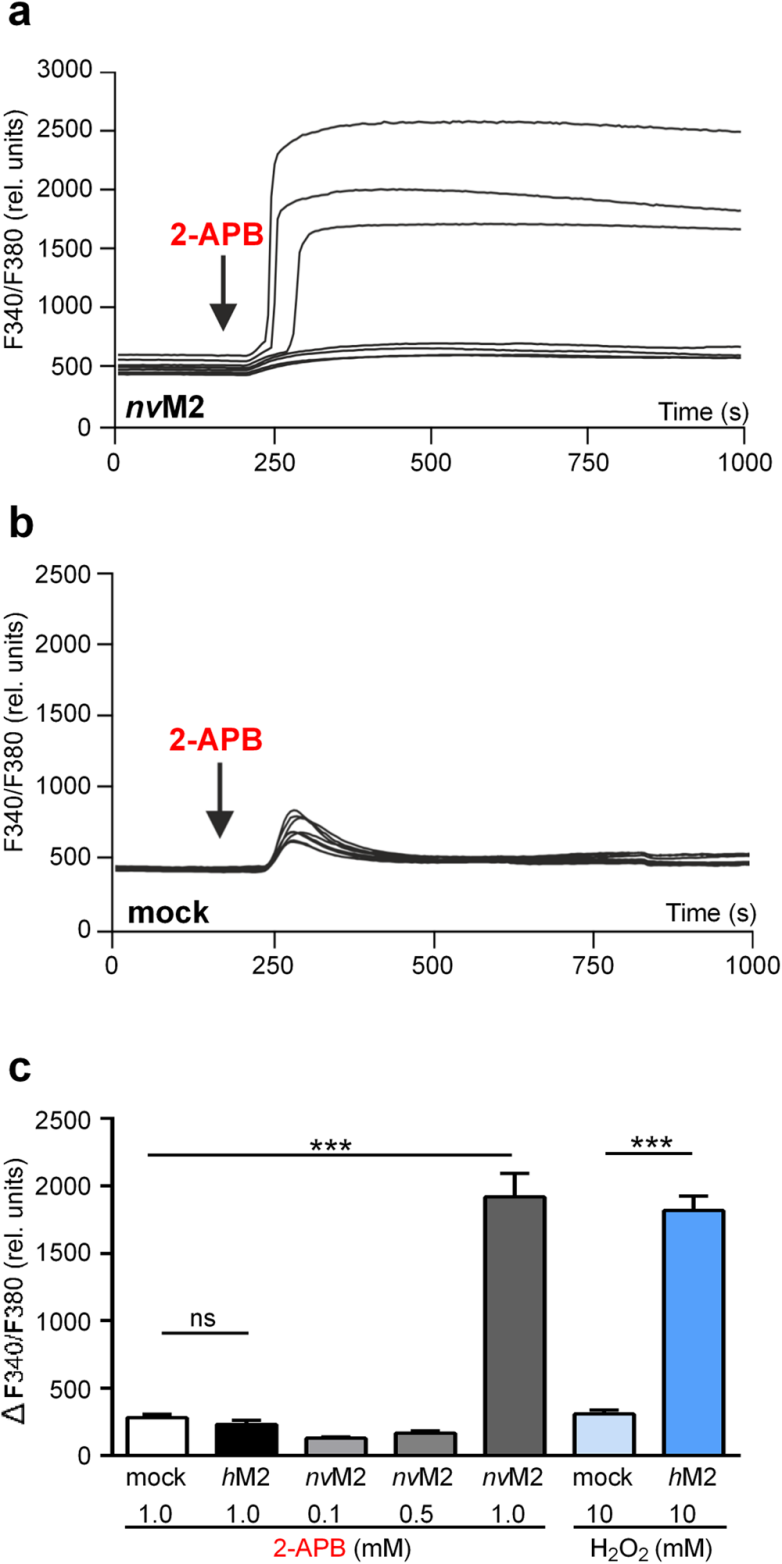
Ca^2+^ entry through *nv*TRPM2 induced by 2-APB in calcium-imaging experiments. (**a**) Representative traces from cells that either did or did not express *nv*TRPM2 after application of 2-APB (1 mM) to the bath. (**b**) Traces from some mock-transfected cells that showed the highest levels of responses that cannot be attributed to 2-APB. (**c**) 2-APB induced Ca^2+^ entry occurs exclusively in cells transfected with *nv*TRPM2 and requires 1 mM 2-APB. Left: Comparison of *nv*TRPM2-transfected cells with mock-transfected and *h*TRPM2-transfected cells (summary). Right: Stimulation of *h*TRPM2 (in the absence of 2-APB) was still possible with H_2_O_2_ (10 mM), demonstrating the functional expression of the channel. Note that the increase of [Ca^2+^]_i_ induced by 2-APB in *nv*TRPM2 is comparable to that of H_2_O_2_ in *h*TRPM2. Asterisks indicates significant differences (*** P < 0.001; Student’s t-test, *n* = 5–15). Error bars are s.e.

Calcium imaging experiments revealed that 2-APB (1 mM) consistently induced long-lasting increases of [Ca^2+^]_i_ in cells transfected with *nv*TRPM2 which were clearly different from the 2-APB effects in mock-transfected control cells (Fig. 4a and b). 2-APB-concentrations of 1 mM were required, whereas no stimulation was observed with lower concentrations (Fig. 4c), confirming the patch-clamp data. Again, the onset of 2-APB effects showed a characteristic delay (30–60 s). Since inhibiting effects of 2-APB may turn into the opposite with high concentrations, as described for *h*TRPM7 (Li *et al*., 2006), we performed calcium imaging experiments on the human orthologue *h*TRPM2 in the presence of 2-APB (1 mM) in the standard bath solution. No stimulation of *h*TRPM2 with this high concentrations of 2-APB (Fig. 4c) was evident. As positive control we used a standard bath solution containing H_2_O_2_ (10 mM) for current stimulation on *h*TRPM2. The characteristic strong increase of [Ca^2+^]_i_ was detected only in cells transfected with *h*TRPM2 but not in mock-transfected cells (Fig. 4c) as previously reported (e.g., ref. 4). To confirm these results in patch-clamp experiments, we used a bath solution with NMDG (140 mM) as sole monovalent cation and with Ca^2+^ (10 mM). In control experiments with ADPR (0.15 mM) as stimulus, we recorded sizable inward currents through wild-type *nv*TRPM2 (Supplementary-Fig. 1a). However, when we used 2-APB (1 mM) for stimulation, no currents were elicited in the NMDG/Ca^2+^ bath solution (Supplementary-Fig. 1b). One possible explanation for this finding is that NMDG prevents the access of 2-APB to the pore. Therefore, we replaced NMDG by sucrose in the bath, with Ca^2+^ (10 mM) still the only cation. This time, the typical currents were evoked by 2-APB (Supplementary-Fig. 1c). This finding demonstrates that 2-APB does not interfere with the permeation of Ca^2+^ through the pore but that NMDG is able to suppress the effect of 2-APB. Similar observations were made on TRPV6 where a direct interaction between NMDG and 2-APB was suggested^25^.

### Mutation of the putative selectivity filter in *nv*TRPM2 changes sensitivity to 2-APB

**Figure 5:**
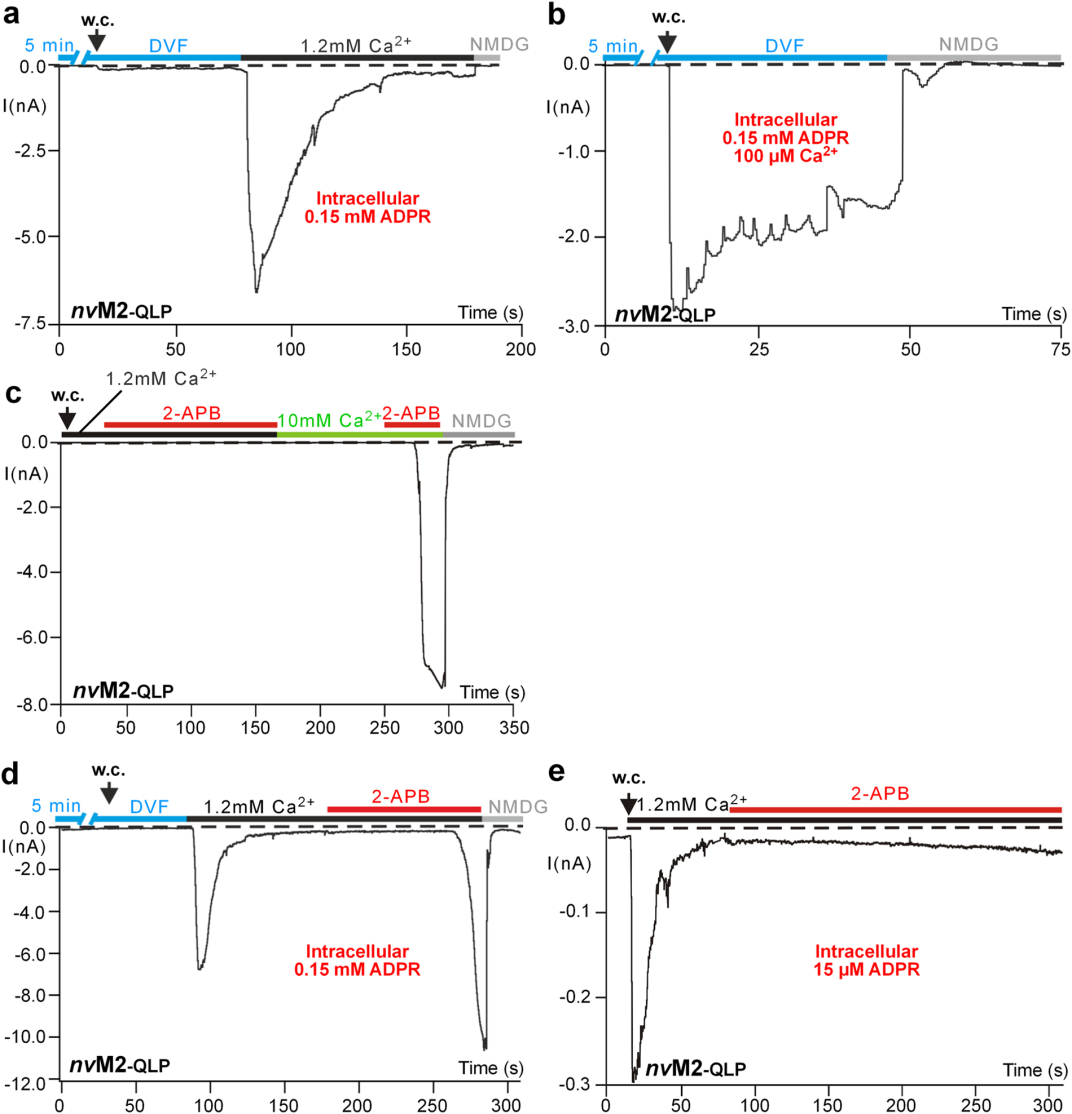
Diminished effects of ADPR and 2-APB in the QLP variant of *nv*TRPM2, restored by elevated Ca^2+^. (**a**) ADPR requires extracellular Ca^2+^ in *nv*TRPM2-QLP in the presence of standard intracellular Ca^2+^ concentrations. With ADPR (0.15 mM) and Ca^2+^ (1 µM) in the pipette solution, current recording was started after extended exposure to DVF. Restoration of standard extracellular Ca^2+^ (1.2 mM) enabled normal currents that were missing without extracellular Ca^2+^. (**b**) ADPR becomes effective on *nv*TRPM2-QLP under extracellular divalent-free conditions when intracellular concentrations of Ca^2+^ are strongly increased. Stimulation was performed with ADPR (0.15 mM) and strongly elevated Ca^2+^ (100 µM) in the pipette solution. Note the decelerated inactivation. (**c**) 2-APB becomes effective in strongly increased concentrations of extracellular Ca^2+^ in *nv*TRPM2-QLP. Two stimulations were performed with 2-APB (1 mM), first in the presence of a standard extracellular Ca^2+^ (1.2 mM) and then at high Ca^2+^ (10 mM). The pipette solution contained Ca^2+^ (1 µM) but no ADPR. (**d**) 2-APB becomes effective in standard extracellular Ca^2+^ (1.2 mM) after pre-stimulation with ADPR. The experiment was started with exactly the same protocol as in panel a, followed by a stimulation with 2-APB (1 mM) that this time did induce currents. Note the long lag time as well as the difference in amplitudes between the two stimulations. 2-APB (1 mM) consistently evoked larger currents than ADPR (0.15 mM) alone. (**e**) Pre-stimulation with low concentrations of ADPR is not sufficient to enable 2-APB effects. With only 15 µM instead of 0.15 mM ADPR in the pipette, small currents were induced but subsequent application of 2-APB (1 mM) failed to evoke the same strong currents as shown in panel c. All experiments were repeated at least three times confirming the results.

To define more closely the site within the pore that governs the Ca^2+^ sensitivity of *nv*TRPM2 to ADPR and/or to 2-APB, we created and studied a variant of *nv*TRPM2 where the original triplet of amino acid residues (ELF) within the proximal pore loop was changed to QLP. We expected that this mutation might reduce access of Ca^2+^ from the outside into the pore. Surprisingly, however, the experimental results were not in agreement with this concept. No stimulation by ADPR was found in the absence of extracellular Ca^2+^ (Fig. 5a) where in wild-type *nv*TRPM2 currents with normal amplitude had been elicited (Fig. 3b). Restitution of extracellular Ca^2+^ to *nv*TRPM2-QLP, on the other hand, immediately enabled intracellular ADPR to induce currents (Fig. 5a). Thus, the QLP variant seems to impede intracellular rather than extracellular Ca^2+^ to act as co-stimulus with ADPR. This interpretation is corroborated by experiments when a strongly elevated intracellular Ca^2+^ concentration (100 µM) was used (Fig. 5b) because then, currents were induced by ADPR. As in wild-type, removal of extracellular Ca^2+^ considerably slowed down the current inactivation (Fig. 5b). Again, there were important differences between the two agonists ADPR and 2-APB with respect to their modification by Ca^2+^. 2-APB completely failed to induce currents in *nv*TRPM2-QLP under standard conditions (1.2 mM extracellular Ca^2+^ and 1 µM intracellular Ca^2+^; Fig. 5c). Nevertheless, there were two conditions where 2-APB-induced currents became possible in the QLP mutant. First, elevation of extracellular Ca^2+^ to 10 mM enabled 2-APB to induce currents with characteristic kinetics (Fig. 5c). Second, when currents had already been stimulated in the cell with ADPR and had been allowed to inactivate completely, the addition of 2-APB (1 mM) to the bath led to large and sustained currents (Fig. 5d).

We performed a statistical comparison of the current amplitudes induced first by ADRP (0.15 mM) and afterwards by 2-APB (1 mM) under these conditions. Consistently, the currents in the presence of 2-APB were considerably larger in amplitude (p < 0.01, *n* = 5, student’s paired-t test), demonstrating that 2-APB truly achieves a channel activation here, rather than only a reversal of inactivation of ADPR-dependent currents. Thus, pre-stimulation of *nv*TRPM2-QLP with ADPR enabled 2-APB to stimulate channels by itself. As demonstrated in Fig. 5e, this effect was quantitatively dependent on the extent of the pre-stimulation with ADPR. When a threshold-concentration of ADPR (≤15 µM) was used that elicited only very small currents in *nv*TRPM2-QLP, the subsequent application of 2-APB was without effect (Fig. 5e).

### Single point mutation within the intracellular S2/S3-linker of *nv*TRPM2 changes sensitivity to 2-APB

**Figure 6:**
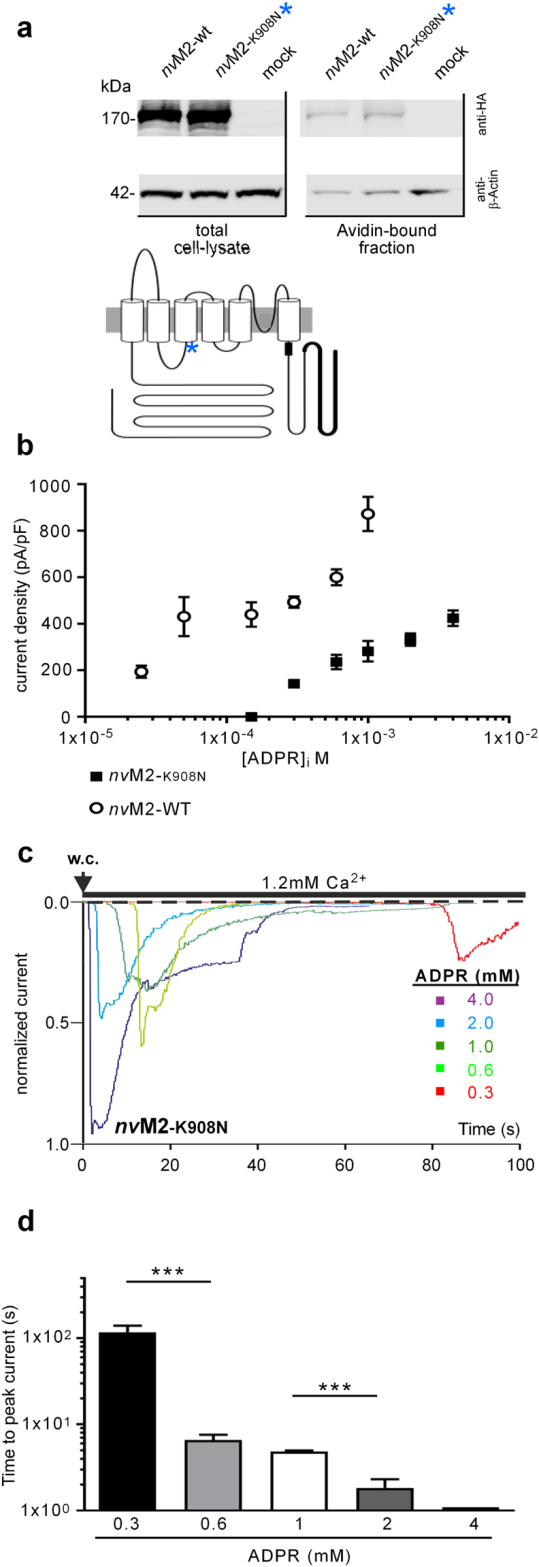
Diminished effects of ADPR in the K908N variant of *nv*TRPM2. (**a**) *Upper*. Cell surface expression, assessed with biotinylation assays, of wild-type *nv*TRPM2 and the variant K908N (as indicated), each containing a C-terminally attached 3xHA tag (only for Western blot analysis). Western blots on the NeutrAvidin-bound fractions (*right*) and on total HEK-293 cell lysates (*left*) were probed with anti-HA antibody. Full-length blots are presented in Supplementary Figure S2. *Lower*. Sketch indicating the approximate location of the point mutation K908N within the *nv*TRPM2 structure. (**b**) On *nv*TRPM2-K908N, ADPR acts with diminished potency and efficacy. Plotted are peak current densities (mean ± S.E.M; *n* = 4–9) of wild-type and mutant, in response to various concentrations of ADPR in the pipette solution (as indicated). (**c**) Current amplitude as well as lag time of current development depend on the ADPR concentrations, as shown in representative traces with various concentrations of ADPR in the pipette solution (as indicated). (**d**) Summary of experiments as derived from panel c. Note the logarithmic ordinate. Significant differences are indicated with asterisks (*** P < 0.001; Student’s t-test, *n* = 5–7). Error bars are s.e.

**Figure 7:**
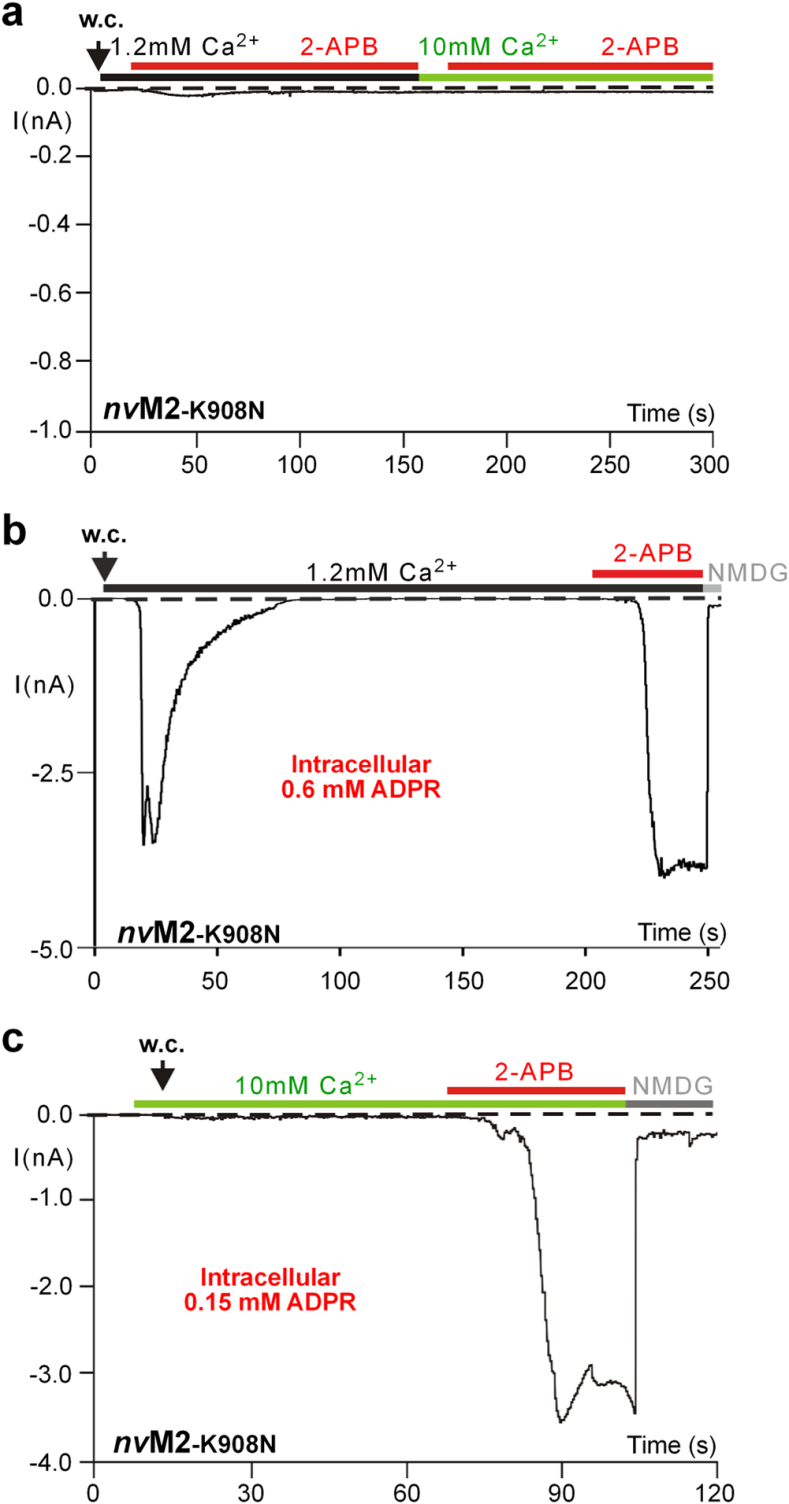
Diminished effects of 2-APB on *nv*TRPM2-K908N. (**a**) 2-APB fails to evoke currents even at elevated extracellular Ca^2+^. (**b**) 2-APB becomes effective after pre-stimulation with ADPR. In contrast to the experiment shown in panel a, ADPR was present in the pipette solution, at a roughly half-maximal concentration (0.6 mM). After complete inactivation of the ADPR-induced current, 2-APB (1 mM) was applied and did evoke sizeable currents. (**c**) 2-APB becomes effective in elevated extracellular Ca^2+^ after pre-stimulation with ADPR at a sub-threshold concentration. Current recording was performed in a bath solution with 10 mM Ca^2+^ and a pipette solution with 0.15 mM ADPR, which did not induce currents in this combination. Currents were only evoked by subsequent application of 2-APB (1 mM) with the typical properties. All experiments were repeated at least three times confirming the results.

After we had analyzed the QLP-variant of *nv*TRPM2 that exhibits a diminished sensitivity to Ca^2+^, we used 2-APB to probe the newly created variant *nv*TRPM2-K908N. This point mutation in the S2-S3 linker, a decisive element for the link between stimulation and pore opening, created channels that were expressed in the plasma membrane indistinguishably to wild-type channels (Fig. 6a) but on which ADPR had dramatically lost potency as well as efficacy (Fig. 6b). Moreover, ADPR needed more time to achieve channel activation, dependent on its concentration (Fig. 6c and d). 2-APB (in the highest attainable concentration of 1 mM) lost its effects as activator on this mutant, and these effects could not be re-gained by elevated Ca^2+^ concentrations in the bath (Fig. 7a). However, similarly as in the QLP variant, 2-APB did induce currents in *nv*TRPM2-K908N when a pre-stimulation with ADPR had been performed. This was possible with ADPR at a concentration of 0.6 mM, which corresponds to a concentration with sub-maximal effects on this variant (Fig. 7b). Moreover, currents were induced by 2-APB in the presence of a sub-threshold concentration of 0.15 mM ADPR but only if the extracellular Ca^2+^ concentration simultaneously had been elevated from 1.2 to 10 mM (Fig. 7c).

## Discussion

The present study reveals that 2-APB activates *nv*TRPM2 in a similar manner as ADPR. For both stimuli, Ca^2+^ serves as an essential co-factor. The different kinetics of current onset shown by the two agonists may reflect the different rates at which Ca^2+^ reaches the activation sites. For ADPR this process occurs instantaneously, whereas for 2-APB at high concentrations, a lag time of several tens of seconds is characteristic. In contrast to ADPR, 2-APB activates from the outside, apparently by penetrating into the pore. Both stimuli enable access of Ca^2+^ to the proposed activation sites in the vicinity of the cytosolic pore entrance^13^. Ca^2+^ is also required for the other major effect of 2-APB, the suppression of current inactivation.

Wild-type *nv*TRPM2 essentially requires Ca^2+^ for its activation by ADPR. When Ca^2+^ is present, full activation by ADPR occurs rapidly, followed by a fast inactivation. Removal of Ca^2+^ by EGTA, a comparably slow complex builder, abolishes ADPR effects, but only when applied on both sides of the plasma membrane. Hence, Ca^2+^ from the outside or from the inside is equally effective. Since rapid activation remains possible when the bath is exchanged to a nominally divalent-free solution, it is concluded that already low amounts of Ca^2+^, characteristic for the cytosol, are sufficient. Moreover, the fact that Ca^2+^ from either side of the cell membrane is effective provides strong evidence that Ca^2+^ acts directly at the pore of the Ca^2+^-permeable channel *nv*TRPM2. This has already been extensively studied and discussed in *h*TRPM2^12, 13^ which in this respect resembles the *Nematostella* orthologue. In contrast to *h*TRPM2 which shows long-lasting currents in response to ADPR, *nv*TRPM2 exhibits fast inactivation kinetics. The inactivation is obviously dependent on Ca^2+^ as well because it is considerably slowed in the long-lasting absence of extracellular Ca^2+^. As molecular explanation, a collapse of the pore is an attractive hypothesis, in line with similar hypotheses on *h*TRPM2^15^.

2-APB has been found in a previous^22^ and in the present study as a reversible inhibitor of *h*TRPM2. In contrast, it is demonstrated in the present study that it is an activator of *nv*TRPM2. Although Ca^2+^ is required for the activation of *nv*TRPM2 by 2-APB as well, there exist a remarkable difference in the Ca^2+^-dependency between 2-APB and ADPR. After addition of 2-APB, a characteristic delay is observed before any effect becomes visible; afterwards, the activation by 2-APB ensues as fast as that by ADPR. Moreover, 2-APB displays a very steep concentration-effect relationship. In combination, the findings are in agreement with a model where 2-APB enters slowly the pore of resting *nv*TRPM2, perhaps during the few spontaneous channel openings that we have demonstrated in single-channel analysis^6^. Only from the outside but not from the inside, access appears to be possible, as already shown for several other TRP channels e.g. *h*TRPM2^22^ and *h*TRPM7^26^. As soon as Ca^2+^ has accumulated in the pore, a full activation takes place rapidly by the combined actions of the two substances that now both have free access to the pore. It seems that an initial threshold must be surpassed which is achieved by 2-APB only at its highest concentration; a self-enhancing process is induced afterwards.

No inactivation occurs in *nv*TRPM2 when 2-APB is the stimulus. In the model attributing inactivation to pore collapse^15^, the pore would remain permanently open in the presence of 2-APB. Remarkably, wash-out of 2-APB lead to an almost immediate reversal of both effects of 2-APB, the channel activation and the prevention of the inactivation. The striking difference between the off-kinetics and on-kinetics are again evidence for an action taking place within the pore and for a much faster diffusion when the pore is open than closed. However, it must remain undecided whether 2-APB is capable of keeping the pore open by itself; alternatively, it may block Ca^2+^ from the site at which the ion mediates pore collapse. The observation that extracellular and intracellular Ca^2+^ must be present when channel stimulation is performed with 2-APB, whereas Ca^2+^ on one side of the cell membrane is sufficient when ADPR is the stimulus, points to higher amounts of Ca^2+^ being necessary for 2-APB than for ADPR. Steric reasons limiting the availability of Ca^2+^ on its regulatory sites may contribute. In the same line, the results with the variant containing the changed pore signature QLP demonstrate that the availability of Ca^2+^ is restricted after this manipulation. It is unexpected that the QLP-variant apparently prevents intracellular Ca^2+^ to become a co-agonist of ADPR, unless huge intracellular Ca^2+^ concentrations are employed. At the same time, the role as co-agonist can still be played by extracellular Ca^2+^. Perhaps one would expect that especially the access from the extracellular side was restrained because the QLP motif reduces the Ca^2+^ selectivity of TRPM channels^3^. Nevertheless, this contradiction may be resolved by taking into consideration the large concentration gradient of Ca^2+^ across the membrane; in a situation where access of Ca^2+^ to regulatory sides is restricted, intracellular Ca^2+^ may be more hampered than extracellular Ca^2+^. Again, the experiments with the QLP-variant reveal that more Ca^2+^ is required for 2-APB than for ADPR because the normal extracellular Ca^2+^ concentration (1.2 mM) was sufficient as co-agonist for ADPR but not for 2-APB, whereas elevation to 10 mM Ca^2+^ restored its capacity.

The K908N mutant of *nv*TRPM2, for the first time introduced and analyzed in the present study, is valuable for the ongoing understanding of ADPR regulation of this channel. In striking contrast to *h*TRPM2, *nv*TRPM2 does not depend on the C-terminal cytosolic NUDT9H domain to be stimulated by ADPR^6^. Most likely, it possesses an alternative binding site for ADPR that may be localized within the N-terminus. In the K908N mutation, dramatically higher concentrations of ADPR are required, by one to two orders of magnitude, than in wild-type *nv*TRPM2. The maximally attainable currents are strongly diminished as well. This makes a diminished binding of ADPR highly unlikely and indicates a disturbed gating process. This interpretation is supported by the general view of the function of the S2-S3 segment in the superfamily of voltage-dependent cation channels that all share a similar structure^27^. In some members of the TRP family that are activated by a combination of electrical and various quite distinct chemical signals, the role of this segment in the gating process has been convincingly demonstrated (e.g. TRPV1, TRPM8^28–31^). Moreover, a corresponding mutation within the S2-S3 segment of Ca_v_1.3 directly affected the coupling between voltage sensor movement and pore opening^32^. Thus, it appears a safe assumption that the K908N mutation impedes the intramolecular communication between the ADPR binding site and the pore within the process of gating.

The 2-APB-dependent gating is disturbed as well in the K908N variant. For this agonist, however, a restoration is not reasonably possible by elevated concentrations because the effective concentration in wild-type channels is already 1 mM. Thus, the positive feed-back between 2-APB and Ca^2+^ cannot be easily initiated, not even by 10 mM Ca^2+^. Interestingly, gating is possible anyway when all three agonists on *nv*TRPM2, i.e. 2-APB, ADPR, and Ca^2+^, are employed in the right combination. The results fit very well into the concept of the dual actions of Ca^2+^ within the channel pore. When the mutant channel is stimulated by ADPR such that a sizable current develops, and is allowed to inactivate by the action of Ca^2+^, subsequent addition of 2-APB evokes currents that exceed in amplitude and duration those initially elicited by ADPR (with ADPR still present on the cytosolic side). Our interpretation is that Ca^2+^ has already entered the pore by ADPR and that now 2-APB can exert its gating effects. Without pre-existing Ca^2+^ in the pore, gating does not take place because the initial threshold is not reached and the co-operation of 2-APB and Ca^2+^ is not started. The notion that threshold phenomena become evident is moreover corroborated by the experiments when ADPR is additionally present, but in concentrations too small to evoke noticeable currents by itself. Then 2-APB is capable of inducing currents only after the extracellular Ca^2+^ concentration is increased to 10 mM.

The usefulness of 2-APB for the present as well as for future experiments is based on several points. First, it is a new stimulus of *nv*TRPM2 which can be applied to the extracellular fluid and exerts effects fully reversible following wash-out. This is a strong advantage over the principal stimulus of *nv*TRPM2, ADPR, which exclusively acts from inside the cell. Thus, ADPR can be employed in patch-clamp experiments but not in usual calcium imaging and its concentration cannot be easily altered during one experiment.

More important than its role as activator, we consider the effects of 2-APB on the inactivation of *nv*TRPM2. The *Nematostella* orthologue exhibits an inactivation that is much faster than in the human counterpart. However, inactivation takes place there as well and may have considerable impact on the biological function of the channel. This is especially evident in the light of a mutation of *h*TRPM2 found in an endemic form of amyotrophic lateral sclerosis and Parkinson’s disease associated dementia. The mutation P1018L induces rapid inactivation in *h*TRPM2 and thus prevents sustained ion influx^14^. The molecular mechanisms of inactivation in TRPM2 channels are far from being understood. The present results indicate that 2-APB interferes with a process that takes place within the pore region of *nv*TRPM2 when inactivation is induced. Specific effects of 2-APB on the permeation pathway have been also described for Orai channels^18, 33^. Further experiments are necessary to elucidate the structural requirements for the inactivation of *nv*TRPM2.

We conclude that 2-APB has a dual effect on *nv*TRPM2, activation and inhibition of inactivation. Both effects take place within the pore and are dependent on Ca^2+2+^. For the future, our study establishes 2-APB as a promising tool that will be useful in elucidating the molecular basis of ADPR-mediated regulation of TRPM2 as well as understanding common and individual principles of gating within the TRPM family.

## Methods

### Molecular cloning

The cDNA of the TRPM2-like channel of *Nematostella vectensis* was synthesized by MWG-Biotech (Ebersberg, Germany) and subcloned as describe elsewhere^4^. The cDNA of *h*TRPM2 was subcloned via *Eco* RI + *Xba* I into the pIRES-hrGFP-2a vector (Stratagene, La Jolla, CA, USA). Site-directed mutagenesis was performed using the QuikChange mutagenesis system (Agilent, Santa Clara, CA, USA). Defined oligonucleotides were obtained from MWG-Biotech. Each point mutation was verified by DNA sequencing (MWG-Biotech). For easy detection in Western blot analysis wild-type and mutant *nv*TRPM2 channels were C-terminally fused with a triple hemagglutinin (3xHA)-tag as described previously^6^. All procedures were performed in accordance to the respective manufacturer’s instructions, unless indicated otherwise.

### Cell culture and transfection

HEK-293 cells were obtained from the German Collection of Microorganisms and Cell Cultures (Braunschweig, Germany) and cultured in DMEM media (Biochrome, Berlin, Germany) supplemented with 4 mM L-glutamine and 10% (v/v) foetal calf serum (Biochrome) and 2 mM sodium pyruvate. Transient transfections of HEK-293 cells with the cDNAs of *h*TRPM2 or *nv*TRPM2 were performed using the FuGene 6 transfection reagent (Roche, Mannheim, Germany) according to the manufacturer’s instructions. As controls, cells were transfected with the pIRES-hrGFP-2a vector alone. The transfected cells were maintained for 24 h in an incubator at 37 °C and 5% CO_2_. Subsequently, the cells were seeded on poly-lysine-coated glass coverslips at a suitable dilution and further incubated for 3–4 h. Then, patch-clamp and calcium imaging experiments were carried out in cells visibly positive for EGFP. At least three independent transfections were used for each experimental group.

### Electrophysiology

Whole-cell recordings were performed using an EPC 9 amplifier equipped with a personal computer with Pulse 8.5 and X Chart software (HEKA, Lamprecht, Germany). The standard bath solution contained (in mM) 140 NaCl, 1.2 MgCl_2_, 1.2 CaCl_2_, 5 KCl, 10 HEPES, pH 7.4 (NaOH). For Na^+^ free solutions, Na^+^ was replaced by 150 mM N-methyl-D-glucamine (NMDG) and the titration was performed with HCl. The divalent free bath solution (DVF) contained (in mM) 150 NaCl, 10 EGTA, 10 HEPES, pH 7.4 (NaOH). The pipette solution contained (in mM) 145 CsCl, 8 NaCl, 2 MgCl_2_, 10 HEPES, pH 7.2 (CsOH) and the Ca^2+^ concentration was adjusted to either ≤10 nM (10 mM Cs-EGTA, no Ca^2+^ addition), 1 µM (0.886 mM Ca^2+^, 1 mM Cs-EGTA) or 100 µM (1.1 mM Ca^2+^ 1 mM Cs-EGTA). In some experiments bath solutions were used containing (in mM) either 140 NMDG or 280 sucrose as well as 10 CaCl_2_ and 10 HEPES, pH 7.4 (NaOH).

The Ca^2+^ concentration of the solutions was calculated using the *MAXC*-program: (http://www.stanford.edu/~cpatton/maxc.html). For the stimulation of TRPM2, Adenosine diphosphate ribose (ADPR, Sigma-Aldrich, Munich, Germany) was prepared as a 100 mM stock solution in distilled water and aliquots kept frozen. ADPR was diluted to the desired concentration in the intracellular (pipette) solutions on the day of the experiment. 2-Aminoethoxy diphenyl borate (2-APB; Sigma-Aldrich) was prepared as 100 mM stock solution in dimethyl sufloxide (DMSO), and aliquots kept frozen. 2-APB. was diluted to the desired concentration in the extracellular solutions on the day of the experiment. Unless otherwise stated, the experiments were performed at room temperature (21 °C) and the current-voltage relations were obtained during voltage ramps from −150 to + 150 mV and back to −150 mV applied over 200 ms. The holding potential was −60 mV. For the analysis the maximal current amplitudes (pA) in a cell were divided by the cell capacitance (pF), a measure of the cell surface. The result is the current density (pA/pF).

### Calcium imaging experiments

For fluorescence imaging of [Ca^2+^]_i_ HEK-293 cells on poly-lysine-coated glass coverslips were loaded in standard bath solution containing membrane-permeable Fura-2 acetoxymethyl ester (1.5 ng/µl; Invitrogen) and pluronic acid (0,025%) for 20 min at 37 °C. Fluorescence was alternately excited at 340 and 380 nm using the Polychrome IV monochromator (TILL Photonics). The emitted fluorescence was measured at 510 nm using a Sensicam (IMAGO). Fluorescence was corrected for background at each wavelength. Measurements were obtained at room temperature (21 °C). The standard bath solution and the application of 2-APB were identical to those described for the patch-clamp experiments. In control experiments currents of *h*TRPM2 were evoked by superfusion of the cells with standard bath solution containing 10 mM H_2_O_2_ (diluted from a 30% stock solution).

### Cell surface biotinylation and Western blot analysis

Biotinylation assays were performed with the Pierce Cell Surface Protein Isolation Kit according to the manufacturer’s instructions (ThermoFisher Scientific, USA). In brief, transfected, sub-confluent HEK-293 cells (90%) were biotinylated and lysed. Samples (600 µg) were incubated with NeutrAvidin beads and a small aliquot of total cell lysate was used as input control. Elution was performed with SDS sample buffer and subjected to SDS-PAGE and Western blot analysis. Detection of β-actin with mouse-anti-β-actin antibody (1:2000; Sigma-Aldrich, USA) and rabbit-anti-mouse-HRP conjugated secondary antibody (1:2000; DAKO A/S, Agilent, USA) was used to rule out that cytosolic proteins in damaged cells were biotinylated. Normalized samples were subjected to reducing SDS-PAGE (4–12% Bis-Tris, NuPAGE, Novex; ThermoFisher Scientific) and Western blot analysis. Expression was determined using a primary monoclonal mouse-anti-HA antibody (1:2000; Sigma-Aldrich) and a rabbit-anti-mouse-HRP conjugated secondary antibody (1:2000; DAKO A/S). Detection was accomplished using the enhanced chemiluminescence detection system (ECL, Amersham Bioscience, USA).

### Data analysis and statistics

Data are expressed as the mean ± s.e.m. Unless statet otherwise, the comparison of two groups was performed using an unpaired Student’s t-test. Calcium imaging experiments were statistically evaluated using a one-way ANOVA and the Bonferroni correction was applied when multiple comparisons were performed with the same control data. Differences were considered significant at **P < 0.01 and ***P < 0.001.

## Electronic supplementary material

Supplementary Information
