# Supplementary material for: Modulation of activation and inactivation by Ca2+ and 2-APB in the pore of an archetypal TRPM channel from Nematostella vectensis
Source: Sci Rep. 2017 Aug 3;7:7245. doi: 10.1038/s41598-017-07652-4 (PMC5543165; doi:10.1038/s41598-017-07652-4)
Supplement: Supplementary file 1 — Supplementary Information [file 41598_2017_7652_MOESM1_ESM.pdf]

Modulation of activation and inactivation by Ca^{2+} and 2-APB in the pore of an archetypal TRPM channel from *Nematostella vectensis*

Frank J.P. Kühn[*], Mathis Winking, Cornelia Kühn, Daniel C. Hoffmann and Andreas Lückhoff

Institute of Physiology, Medical Faculty, RWTH Aachen, D52057 Aachen, Germany

Supplementary-Information

Supplementary-Figure legends

Figure S1. NMDG suppresses the effect of 2-APB in whole-cell patch-clamp experiments

(a) ADPR induces permeation of Ca^{2+} through the pore of *nv*TRPM2. The experiment was performed in a bath solution containing NMDG (140 mM) and Ca^{2+} (10 mM). Stimulation was performed with ADPR (0.15 mM) and Ca^{2+} (1 µM) in the pipette solution. **(b)** NMDG suppresses the effects of 2-APB. The bath solution was initially the same as in panel a, but the pipette solution contained no ADPR and stimulation was performed with 2-APB (1 mM in the bath). Note that the characteristic currents were induced only after substitution of the NMDG-bath solution to standard bath solution containing Na^{+} (140 mM) and Ca^{2+} (1.2 mM). **(c)** 2-APB enables permeation of Ca^{2+} through the channel pore. The experimental conditions were as in panel b, but with sucrose in the bath instead of NMDG. Note that 2-APB induced large inward currents, in spite of the relative low concentration of the only charge carrier, i.e. Ca^{2+} (10 mM). All experiments were repeated at least three times confirming the results.

Figure S2. Full length Western-Blot of Biotinylation experiment depicted in Figure 6a

Note that the additional lane for human TRPM2 (*h*M2-wt) is omitted in Figure 6a since its expression does not belong to the immediate context of Figure 6.

Figure-S1

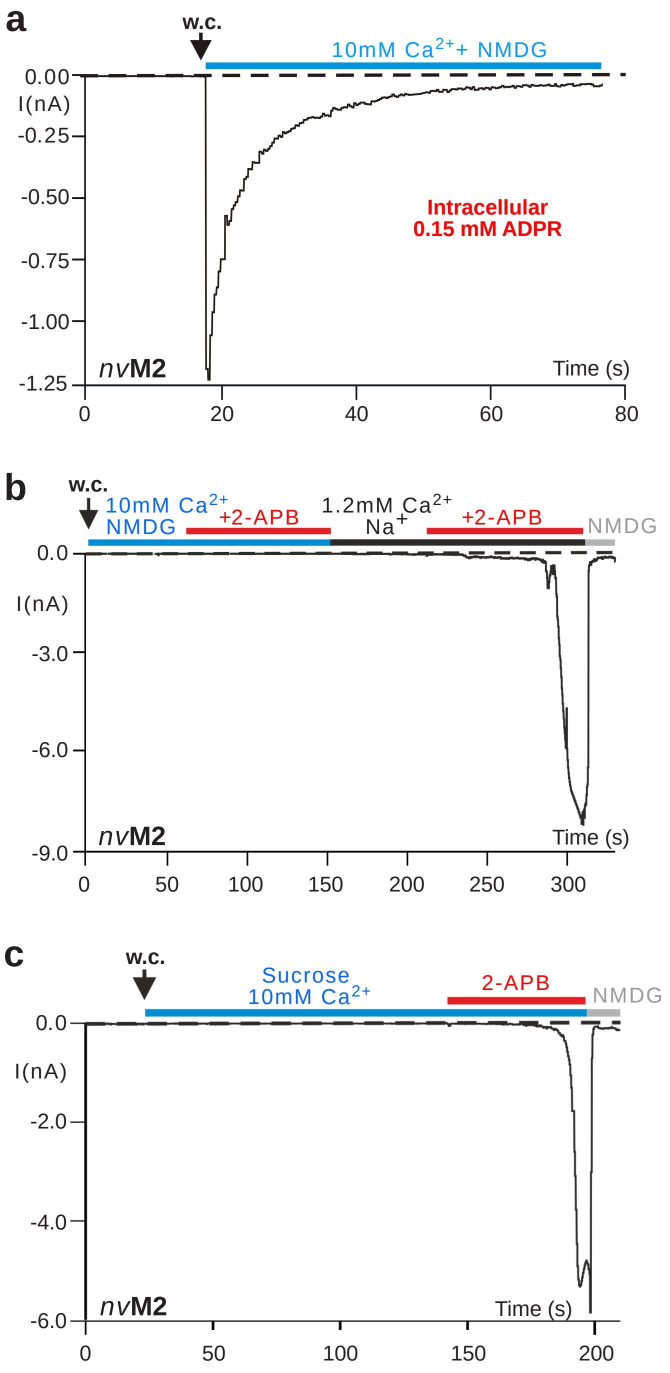
a
w.c.
10mM Ca2++ NMDG
0.00
I(nA)
-0.25
-0.50
-0.75
-1.00
-1.25
Intracellular
0.15 mM ADPR
nvM2
Time (s)
0
20
40
60
80
b
w.c.
10mM Ca2+
NMDG
+2-APB
1.2mM Ca2+
Na+
+2-APB
NMDG
0.0
I(nA)
-3.0
-6.0
-9.0
nvM2
Time (s)
0
50
100
150
200
250
300
c
w.c.
Sucrose
10mM Ca2+
2-APB
NMDG
0.0
I(nA)
-2.0
-4.0
-6.0
nvM2
Time (s)
0
50
100
150
200

Figure-S2

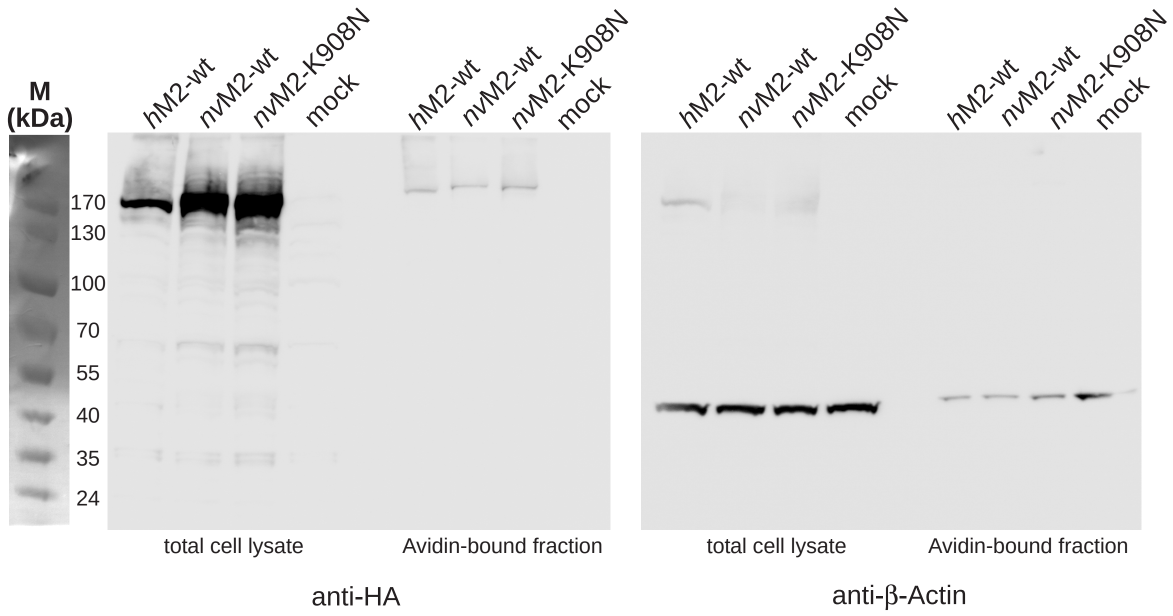
M
(kDa)
hM2-wt
nvM2-wt
nvM2-K908N
mock
hM2-wt
nvM2-wt
nvM2-K908N
mock
hM2-wt
nvM2-wt
nvM2-K908N
mock
hM2-wt
nvM2-wt
nvM2-K908N
mock
170
130
100
70
55
40
35
24
total cell lysate
Avidin-bound fraction
total cell lysate
Avidin-bound fraction
anti-HA
anti-β-Actin
